# Determination of Fire Parameters of Polyamide 12 Powder for Additive Technologies

**DOI:** 10.3390/polym13173014

**Published:** 2021-09-06

**Authors:** Richard Kuracina, Zuzana Szabová, Eva Buranská, Alica Pastierová, Peter Gogola, Ivan Buranský

**Affiliations:** 1 Institute of Integral Safety, Faculty of Materials Science and Technology in Trnava, Slovak University of Technology in Bratislava, Ul. Jána Bottu 2781/25, SK-917 24 Trnava, Slovakia; eva.buranska@stuba.sk (E.B.); alica.pastierova@stuba.sk (A.P.); 2 Institute of Materials, Faculty of Materials Science and Technology in Trnava, Slovak University of Technology in Bratislava, Ul. Jána Bottu 2781/25, SK-917 24 Trnava, Slovakia; peter.gogola@stuba.sk (P.G.); ivan.buransky@stuba.sk (I.B.)

**Keywords:** minimum ignition temperature of dispersed dust, dust explosion, dust cloud, polyamide 12, additive technologies

## Abstract

The use of additive technologies keeps growing. Increasingly, flammable powder materials are also used in additive technologies, and there is a risk of explosion or fire when using them. The current article deals with the determination of fire parameters of a powder sample of polyamide Sinterit PA12 Smoth in accordance with the EN 14034 and EN ISO/IEC 80079-20-2 standards. For that purpose, a sample at a median size of 27.5 µm and a humidity of 0% wt. was used. The measurements showed that the maximum explosion pressure of the PA12 polyamide sample was 6.78 bar and the value of the explosion constant K_st_ was 112.2 bar·m·s^−1^. It was not possible to determine the MIT value of the settled dust, since the melting point of polyamide sample is low. The MIT of the dispersed dust was 450 °C. Based on the measured results, it can be stated that the powdered polyamide PA12 poses a risk in terms of explosions and fires. Therefore, when using polyamide PA12 in additive technologies, it is necessary to ensure an effective explosion prevention.

## 1. Introduction

Additive manufacturing (AM) is gaining increasing importance in industry, not just as a technology for prototyping, but also, and in most cases, for production of functional parts in various fields. Polyamides, or nylons, were the first materials to be recognized as engineering thermoplastics, owing to their superior mechanical properties (especially when exposed to elevated temperatures or solvents) [1,2,3,4,5].

Additive manufacturing is defined by ISO 17296 and ASTM F2792 as “the process of joining materials to make parts or objects from 3D model data, usually layer upon layer, as opposed to subtractive manufacturing methodologies” [6]. Three of the basic types of AM used laser for building up the parts, wherein the starting materials used in the AM laser processes may be in the form of a powder [7].

Dust explosion poses a risk for the processes where dispersed dust occurs. It is therefore necessary to apply the principles of effective explosion protection [8,9,10,11].

In the case of powdered polymers, it is possible to design suitable explosion prevention measures based on the measurement of fire characteristics (minimum ignition temperature of settled and agitated dust (MIT), maximum pressure rise rate, explosion constant (Kst) and lower explosion limit).

There are several databases and literature sources dealing with the explosiveness of dispersed dusts [12,13,14,15,16]. In the field of polyamide polymer powders, only information on nylon 6 and 6,6 fibrous materials can be found. Although the amount of polyamide PA12 used has increased significantly, information on its hazards is quite limited (MIE in [17]), and we have not encountered the research into the explosion parameters of polyamide PA12 so far.

Polyamide PA12 is being increasingly used in industry in additive technologies. It is therefore necessary to know its fire characteristics of MIT [18] and explosion parameters. The PA12 powder is handled in relatively large quantities (additive technologies), and the initiating source for the explosion of dispersed dust is almost always present in these technologies (laser, high temperature...).

## 2. Materials and Methods

### 2.1. Sinterit PA12 Smooth Powder

**Figure 1:**
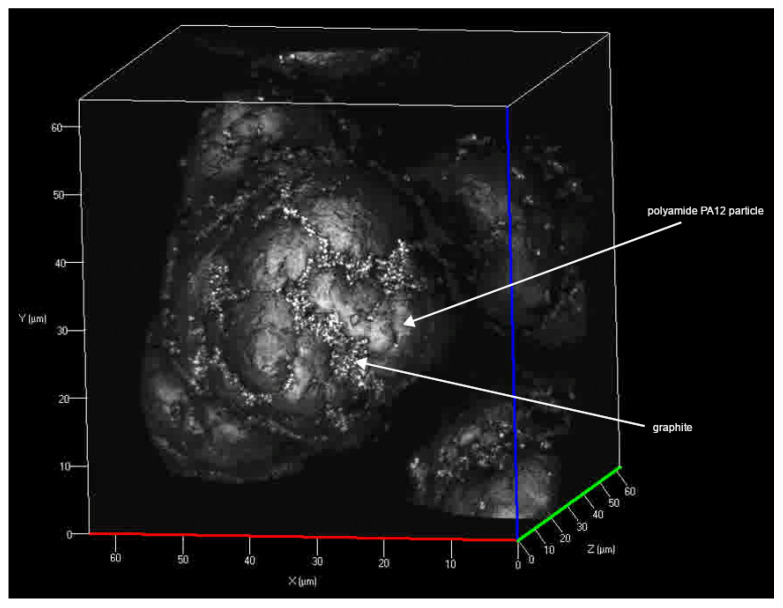
Particles of “Sinterit PA 12 smooth” for laser sintering (confocal laser scanning microscope).

Sinterit PA12 Smooth polymer polyamide powder was used in this study. According to MSDS, the melting point of PA12 is 182 °C [19]. The moisture content of the PA 12 sample is 0% wt. The proportion of dimensional fractions of the PA12 polyamide sample was determined by sieve analysis. The analysis procedure was performed according to the ISO 3310-1:2016 (25 9610) Standard. The analysis was conducted on a Retsch AS 200 sieving machine with a sieving time of 15 min and an amplitude of 2 mm/G. The results of the PA12 sample sieve analysis are given in Table 1. The median value of the sample was 27.5 μm. The particle shape of the PA12 powder is shown in Figure 1.

Topography of powder particles was documented using a ZEISS LSM700 scanning confocal microscope. 405 nm light source was used which in combination with a Epiplan-Apochromat 100x/0.95 objective enabled to reach step sizes of 110 nm on the X and Y axis as well as 60 nm in the Z axis, Figure 1.

**Figure 2:**
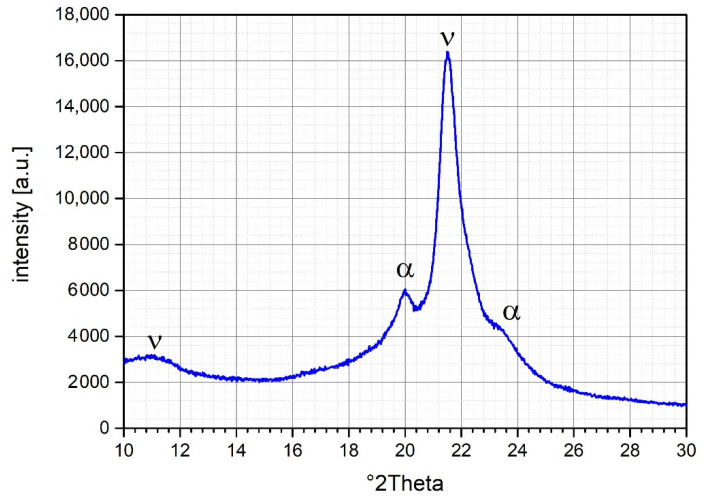
X-ray diffraction patterns of PA12 sample PA 12.

The X-ray diffraction measurements were performed using a Panalytical Empyrean diffractometer. The Cu–Kα nickel filtered radiation was detected in the range of 10–140° 2Theta. Figure 2 shows the XRD pattern for the investigated PA-12 powder. Good agreement with related publications was found. In the current powder both crystalline α and γ phases were observed with their corresponding peaks. Peaks at 20° and 23° 2theta correspond to the α phase. Peaks at 11.2° and 21.5° 2theta correspond to the γ phase [20,21,22,23,24].

For FTIR (FT-IR Spectrometer 660, Varian, Palo Alto, CA, USA) analysis, samples of sheaths were directly applied to a diamante crystal of ATR (GladiATR, Pike Technology, Fitchburg, WI, USA). The spectra were recorded using a Varian Resolutions Pro, and the samples were measured in the region of 400–4000 cm^−1^; each spectrum was measured 48 times, at the resolution of 4.

**Figure 3:**
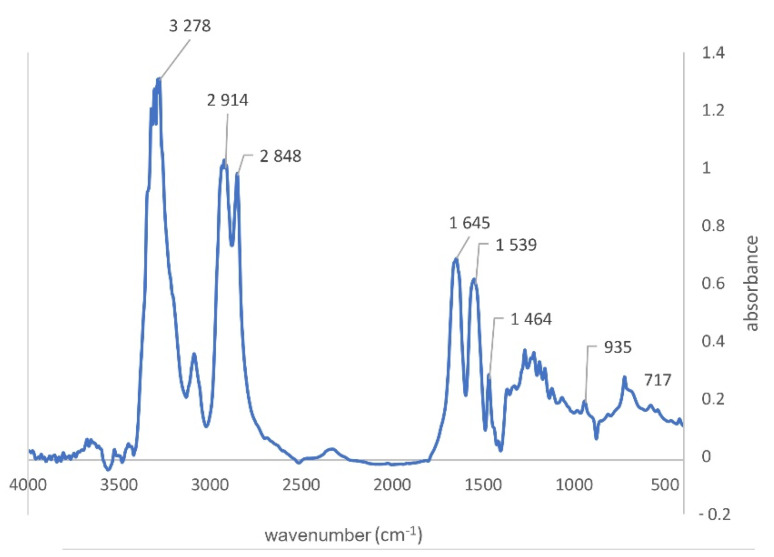
Infrared spectra of PA12 sample.

Figure 3 shows the infrared spectra of PA12. The infrared spectrum of the sample is typical for polyamide. The characteristic bands at a wavelength of 3278 cm^−1^ and 1645 cm^−1^ are attributed to the bending and stretching –NH_2_ bond. In addition, the vibration at 1650 cm^−1^ corresponds to the –CO, and the band at 1540 cm^−1^ is due to the -NH deformation and -CN of the secondary amides. The peaks between 2800 and 2900 cm^−1^ owing to the presence of -CH_2_, -CH_3_ and NH; the bands at 720, 1470 and 1539–1645 cm^−1^ are assigned to the bonds N–H, C–CO–NH_2_ and C=O of the primary amide.

### 2.2. MIT of Dispersed Dust

**Figure 4:**
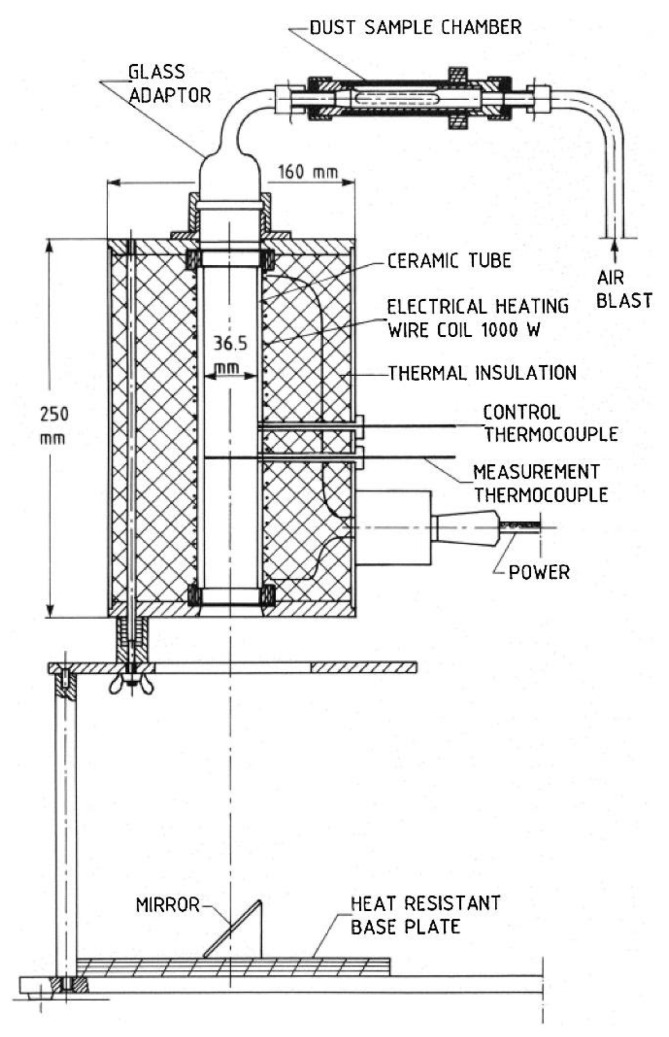
Cross-section of Godbert-Greenwald furnace [25].

**Figure 5:**
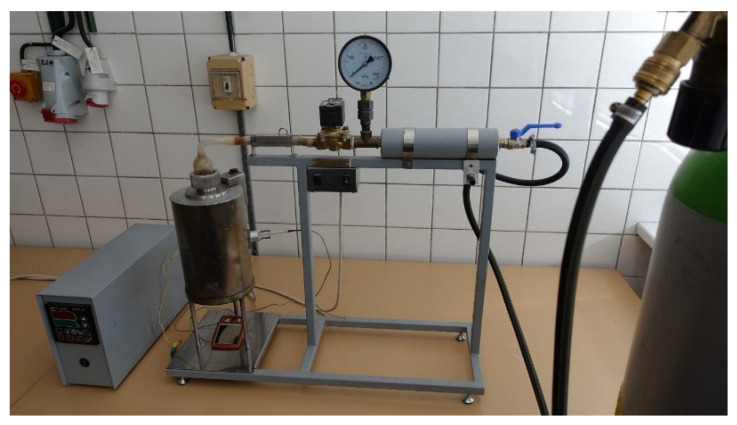
G-G furnace for determination of MIT of dispersed dust.

The MIT measurements of dispersed dust were performed on a standardized equipment, Godbert-Greenwald furnace, Figure 4 and Figure 5 [25].

Small quantities of dust are blown vertically downward through a heated furnace, and ignition is detected by visual inspection. The test material is dispersed in the furnace by air blast. Since most of the sample consists of two fractions (>32 µm, >45 µm), the resulting MIT value depends mainly on these two fractions. Owing to their percentage in the sample, other fractions have a negligible effect on the MIT value of the dispersed dust from the hot surface.

The dust quantity is 0.15 g (corresponds to the concentration with the highest P_max_ value) and the dust is dispersed at the air pressure 20 kPa and 50 kPa. If a burst of flame is seen below the end of the furnace tube, this shall be considered as an ignition. For each combination of temperature and pressure, five measurements were performed. The measurements were assessed “YES” if at least one test was performed with positive results. MIT of the dispersed dust is recorded as the lowest temperature of the furnace at which ignition was obtained, minus 20 K [26].

Owing to the dust characteristics according to MSDS (melting point 182 °C), the MIT determination of settled dust was not performed.

### 2.3. Explosion Parameters

**Figure 6:**
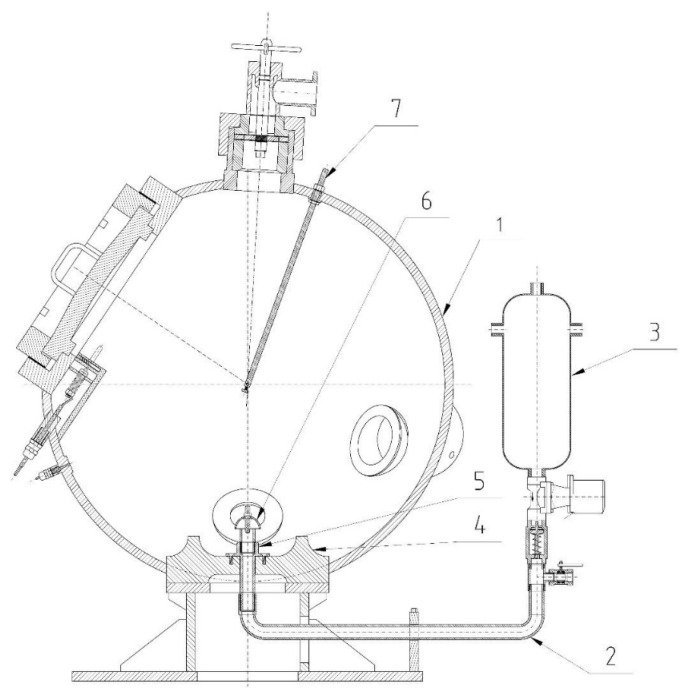
Cross-section of the KV 150M2 explosion chamber (1, chamber; 2, disperser tube; 3, air pressure vessel; 4, dispersing plate; 5, disperser; 6, air flow reverser; 7, igniter rod).

**Figure 7:**
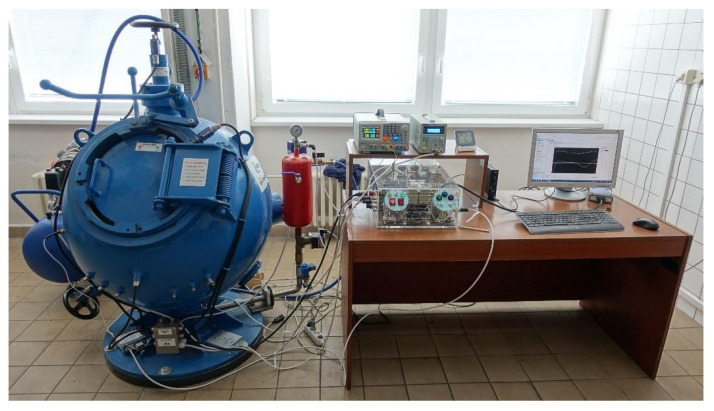
KV 150M2 explosion chamber.

The explosion parameters of PA12 polyamide were determined in the KV 150M2 explosion chamber, Figure 6 and Figure 7.

A KV 150-M2 explosion chamber was used to determine the explosion parameters of PA12 polyamide. Compressed air for dispersing the dust is supplied from a compressed air vessel (6.5 L at 10 bar) through a fast-opening valve into the chamber. The volume of the chamber is 365 L. The sample is placed on a disperser plate and is dispersed by a stream of compressed air. The sample is then ignited by an igniter with an energy of 2 × 5 kJ. The igniter is located in the middle of the explosion chamber according to the EN 14034 Standard [27].

The time between opening the dispersing valve and activation of the igniter is 350 ms. Pressure changes inside the chamber are recorded by pressure transducers. The Keller pressure transmitter has a response of 2000/s, the Kulite pressure transmitter has a response of 410,000/s. The values are recorded at a speed of 50,000/s. Pressure changes during the explosion of dust clouds were measured at the following concentrations: 15 g·m^−3^, 30 g·m^−3^, 60 g·m^−3^, 125 g·m^−3^, 250 g·m^−3^, 500 g·m^−3^, 750 g·m^−3^ and 1000 g·m^−3^.

The measurement was performed three times at each concentration. The P_max_ value is the highest value obtained during the measurements. d*P*/d*t* values were obtained by deriving a smoothed P-t curve (FFT filter, 200 Hz = 125 points of window).

## 3. Results

The minimum hot surface temperature (MIT) causing ignition of dispersed dust was determined in a G-G furnace. The measured values are listed in Table 2.

**Figure 8:**
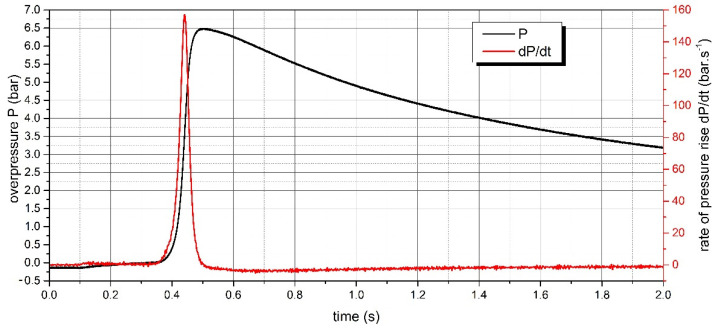
Pressure record of PA12 polyamide explosion at concentration 500 g·m^−3^.

**Figure 9:**
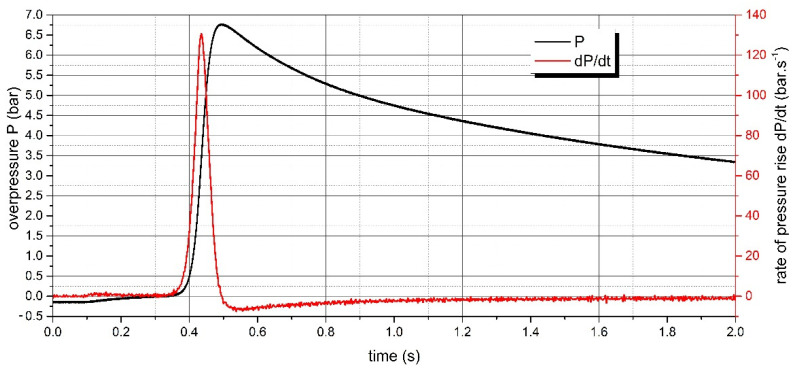
Pressure record of PA12 polyamide explosion at concentration 750 g·m^−3^.

**Figure 10:**
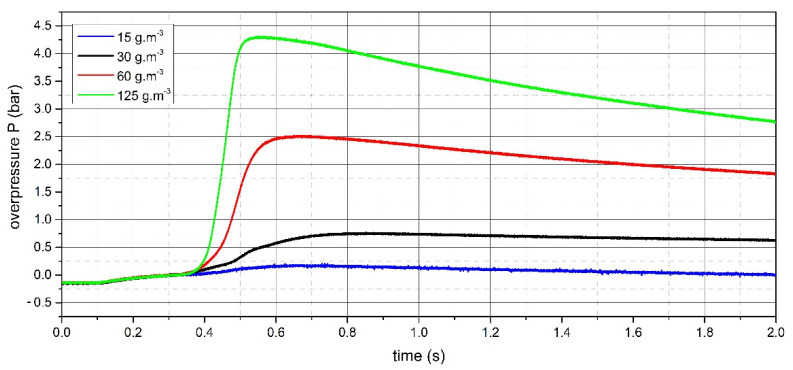
Pressure records for the LEL determination of PA12 polyamide sample.

The measurement of the explosion parameters of dispersed dust sample of PA 12 polyamide was performed in the KV 150M2 explosion chamber. The pressure record of the measurement for the concentration of 500 g·m^−3^, where the highest value of d*P*/d*t* was reached, is shown in Figure 8. The pressure record for the concentration of 750 g·m^−3^, where the highest value of Pmax was reached, is given in Figure 9. The pressure records important for determining LEL are recorded in Figure 10. All measured values are listed in Table 3.

The value of constant explosion *K*_st_ of the sample Sinterit PA12 Smooth was calculated as follows:

(1) $$K_{st}=\sqrt[{3}]{V}\times {\left(dP/dt\right)}_{max}=\sqrt[{3}]{0.365}\times 157.0=112.2\;\mathrm{bar}\cdot s^{-1}\cdot m$$

## 4. Discussion

In our research, the explosion parameters and MIT of dispersed dust of PA12 polyamide were measured. The highest value of explosion overpressure of 6.78 bar for the sample was measured at a concentration of 750 g·m^−3^. The value of the explosion constant was 112.2 bar·m·s^−1^. The MIT value for dispersed PA12 polyamide dust is 450 °C. As shown in Figure 10, the LEL of the Sinterit PA12 Smooth sample is 15 g·m^−3^.

It is not possible to compare the measured results of this particular sample with the measurement results of identical sample attained by other researchers; measurements of explosion parameters of this type of explosive sample (Sinterit PA 12 Smooth) have not been published yet. Generally, only parameters of PA12 polyamide fibers and flocks from the textile industry are available. For the sample “Polyamide flock” of the mean particle size 37 µm, Field [13] states the value of MIT (dispersed dust) 520 °C, the value P_max_ = 9.8 bar and the value K_st_ = 93 bar·m·s^−1^ and LEL = 30 g·m^−3^. Iarossi et al. [28] determined P_max_ = 6.6 bar, K_st_ = 50 bar·m·s^−1^ and MIT (dispersed dust) 485 °C for the fibers with a length of 0.5 mm and dtex 1.7. For polyamide (7367) with a sample median size of 30 µm, the Gestis database [29] determined LEL = 30 g·m^−3^, P_max_ = 7.3 bar, explosion constant K_st_ = 117 bar·m·s^−1^ and MIT (dispersed dust 480 °C). It can therefore be stated that the measured values that Sinterit PA12 Smooth fire properties are comparable with the results indicated in the scientific literature and databases.

## 5. Conclusions

Frequently utilized for the advantages they provide, additive technologies widely use powder materials, including PA12 polyamide (Sinterit PA12 Smooth). It is therefore necessary to deal which safety when using polyamide PA12 in additive technologies. One of the PA12 polyamide is its explosiveness. Explosion and ignition of dispersed dust can cause damage to property and lives.

Based on these values, it can be concluded that the use of PA12 in additive technologies can pose a significant risk of explosion or fire. When using PA12 polyamide in additive technologies, it is therefore necessary to ensure effective explosion prevention.

## Figures and Tables

**Table 1:** Proportion of particle sizes in the sample.

Mesh Size [μm]	Proportion of Particles Sizes in the Sample [%]
500	0.02
250	0.08
200	0.02
150	0.06
90	0.88
71	1.78
56	3.36
45	15.49
32	74.92
20	3.39
0	0.00
**median:**	**27.5 μm**

**Table 2:** Measured values of MIT (sample weight 0.15 g).

Air Pressure [kPa]	Temperature [°C]	Result
20	400	NO
20	410	NO
50	420	NO
50	430	NO
50	440	NO
50	450	NO
50	460	**YES**
50	470	**YES**
50	465	**YES**

**Table 3:** Explosion parameters of PA12 polyamide sample.

Concentration [g·m^−3^]	P_max_ [bar]	d*P*/d*t* [bar·s^−1^]
30	0.75	4.4
60	2.52	23.6
125	4.31	54.4
250	5.67	109.0
500	6.49	**157.0**
750	**6.78**	130.7
1000	6.33	107.2

## Data Availability

The data presented in this study are available on request from the corresponding author.

## References

[1] Cicala G., Latteri A., Del Curto B., Russo A.L., Recca G., Farè S. (2017). Engineering Thermoplastics for Additive Manufacturing: A Critical Perspective with Experimental Evidence to Support Functional Applications. J. Appl. Biomater. Funct. Mater..

[2] Peters E.N., Kutz M. (2017). 1—Engineering Thermoplastics—Materials, Properties, Trends. Applied Plastics Engineering Handbook.

[3] Gibson I., Rosen D., Stucker B., Mahyar K. (2021). Additive Manufacturing Technologies.

[4] Martynková G.S., Slíva A., Kratošová G., Barabaszová K., Študentová S., Klusák J., Brožová S., Dokoupil T., Holešová S. (2021). Polyamide 12 Materials Study of Morpho-Structural Changes during Laser Sintering of 3D Printing. Polymers.

[5] Schneider K., Wudy K., Drummer D. (2020). Flame-Retardant Polyamide Powder for Laser Sintering: Powder Characterization, Processing Behavior and Component Properties. Polymers.

[6] ASTM F2792-12a (2013). Standard Terminology for Additive Manufacturing Technologies.

[7] Schmidt M., Merklein M., Bourell D., Dimitrov D., Hausotte T., Wegener K., Overmeyer L., Vollertsen F., Levy G.N. (2017). Laser based additive manufacturing in industry and academia. CIRP Ann..

[8] Hassan J., Khan F., Amyotte P., Ferdous R. (2014). Industry specific dust explosion likelihood assessment model with case studies. J. Chem. Health Saf..

[9] Dobashi R. (2017). Studies on accidental gas and dust explosions. Fire Saf. J..

[10] Eckhoff R. (1996). Prevention and mitigation of dust explosions in the process industries: A survey of recent research and development. J. Loss Prev. Process. Ind..

[11] Fumagalli A., Derudi M., Rota R., Copelli S. (2016). Estimation of the deflagration index K St for dust explosions: A review. J. Loss Prev. Process. Ind..

[12] Amyotte P.R., Pegg M.J., Khan F.I., Nifuku M., Yingxin T. (2007). Moderation of dust explosions. J. Loss Prev. Process. Ind..

[13] Field P. (1982). Dust Explosions.

[14] Amyotte P.R. (2014). Some myths and realities about dust explosions. Process. Saf. Environ. Prot..

[15] Amyotte P.R., Cloney C.T., Khan F.I., Ripley R.C. (2012). Dust explosion risk moderation for flocculent dusts. J. Loss Prev. Process. Ind..

[16] Addo A., Dastidar A.G., Taveau J.R., Morrison L.S., Khan F.I., Amyotte P.R. (2019). Niacin, lycopodium and polyethylene powder explosibility in 20-L and 1-m3 test chambers. J. Loss Prev. Process. Ind..

[17] Bernard S., Youinou L., Gillard P. (2013). MIE determination and thermal degradation study of PA12 polymer powder used for laser sintering. J. Loss Prev. Process. Ind..

[18] Półka M., Salamonowicz Z., Wolinski M., Kukfisz B. (2012). Experimental Analysis of Minimal Ignition Temperatures of a Dust Layer and Clouds on a Heated Surface of Selected Flammable Dusts. Procedia Eng..

[19] (2021). Sinterit, PA12 Smooth—Technical Datasheet. https://seeda.nl/wp-content/uploads/2018/10/PA12_SMOOTH.pdf.

[20] Salmoria G.V., Paggi R.A., Lago A., Beal V.E. (2011). Microstructural and mechanical characterization of PA12/MWCNTs nanocomposite manufactured by selective laser sintering. Polym. Test..

[21] Ishikawa T., Nagai S., Kasai N. (1980). Effect of casting conditions on polymorphism of nylon-12. J. Polym. Sci..

[22] Liu Y., Zhu L., Zhou L., Li Y. (2019). Microstructure and mechanical properties of reinforced polyamide 12 composites prepared by laser additive manufacturing. Rapid Prototyp. J..

[23] Androsch R., Stolp M., Radusch H.-J. (1996). Simultaneous X-ray diffraction and differential thermal analysis of polymers. Thermochim. Acta.

[24] Schmid M., Kleijnen R., Vetterli M., Wegener K. (2017). Influence of the Origin of Polyamide 12 Powder on the Laser Sintering Process and Laser Sintered Parts. Appl. Sci..

[25] Eckhoff R.K. (2019). Origin and development of the Godbert-Greenwald furnace for measuring minimum ignition temperatures of dust clouds. Process. Saf. Environ. Prot..

[26] (2016). EN ISO/IEC 80079-20-2. Explosive Atmospheres—Part 20—2: Material Characteristics—Combustible Dusts Test Methods.

[27] (2011). STN EN 14034+A1. Determination of Explosion Characteristics of Dust Clouds.

[28] Iarossi I., Amyotte P.R., Khan F.I., Marmo L., Dastidar A.G., Eckhoff R.K. (2013). Explosibility of polyamide and polyester fibers. J. Loss Prev. Process. Ind..

[29] GESTIS Dust Ex Database. https://staubex.ifa.dguv.de/explokomp.aspx?nr=7367&lang=e.

